# The co-evolution of hominins and Stone Age culture

**DOI:** 10.1098/rspb.2026.0772

**Published:** 2026-10-14

**Authors:** Alastair Key

**Affiliations:** Department of Archaeology, Cambridge University, Cambridge, UK

**Keywords:** stone tools, dual inheritance, gene-culture co-evolution, *Homo*, African archaeological record, *Australopithecus*

## Abstract

Human biological and cultural evolution are tightly intertwined; the two display dependent relationships and each influences the trajectory of the other. Archaeological evidence for hominin culture first emerges *ca* 3.3 million years ago, but increasing data attests to an earlier, deeply rooted onset of gene-culture co-evolution in our lineage. Diverse phenotypic traits are linked to Stone Age culture via the latter's impact on the survival and success of early human populations. Hominin cognitive and language capabilities, along with the anatomy of the hand, are key examples. Similarly, variation in Stone Age culture can be linked to the biological traits of the populations from which it derives; important examples concern stone technologies, hunting strategies and social organization. The co-evolution of hominins and Stone Age culture is, therefore, complex, multi-factorial and one of the major stories in human origins research. Here, current understanding of hominin cultural and biological co-evolution prior to the advent of agriculture is reviewed, and new links between the fossil and archaeological records are drawn.

## 1. Introduction

Human culture is uniquely complex and diverse. The archaeological record provides evidence of how our cultural complexity and diversity accumulated through the Plio-Pleistocene [1]. *Homo sapiens* are also unique in the strength of their dependence on cultural knowledge for survival; our Pleistocene ‘generalist specialist’ niche partly relied on a population's cumulative cultural knowledge [2]. Gene-culture co-evolutionary (dual inheritance) mechanisms have also uniquely [3] impacted human evolution, with a diverse array of traits linked to the tightly intertwined relationship between our culture and biology [4–6]. The longevity and strength of our dependence on complex cultural traits suggest this co-evolutionary relationship is deeply rooted.

Gene-culture co-evolution (GCC) has come to the fore of human origins research in the last 25 years. A wealth of advances has vastly expanded our understanding beyond the broad relationships outlined by Ambrose [7]. Fundamentally, GCC is a reciprocal mechanism where a population's biological traits impact the evolutionary trajectory experienced by their cultural traits, and *vice versa* [8]. The adoption of a given cultural variant may disproportionately increase the fitness of some individuals within a larger population, meaning any genetic information linked to that increase becomes disproportionately represented in subsequent generations. In turn, later populations will more efficiently and/or effectively enact the cultural variant, potentially increasing their dependence on it (and therefore rates of copying) and/or impacting how they express it via cultural evolutionary mechanisms [9]. Subsequent cultural variants may, in turn, perform a given task more effectively and/or efficiently and be preferentially replicated, further increasing a population's cultural dependence and, again, disproportionately increasing the fitness of some individuals within a population. Ultimately, a co-evolutionary loop can develop, whereby biological and cultural traits impact the evolution of the other over extended generations (figure 1). Diverse reviews outline the mechanisms underpinning GCC, and ultimately niche construction, in humans and other apes [4,6,8,10–12], including those directed towards archaeological evidence [5,13].

**Figure 1.**
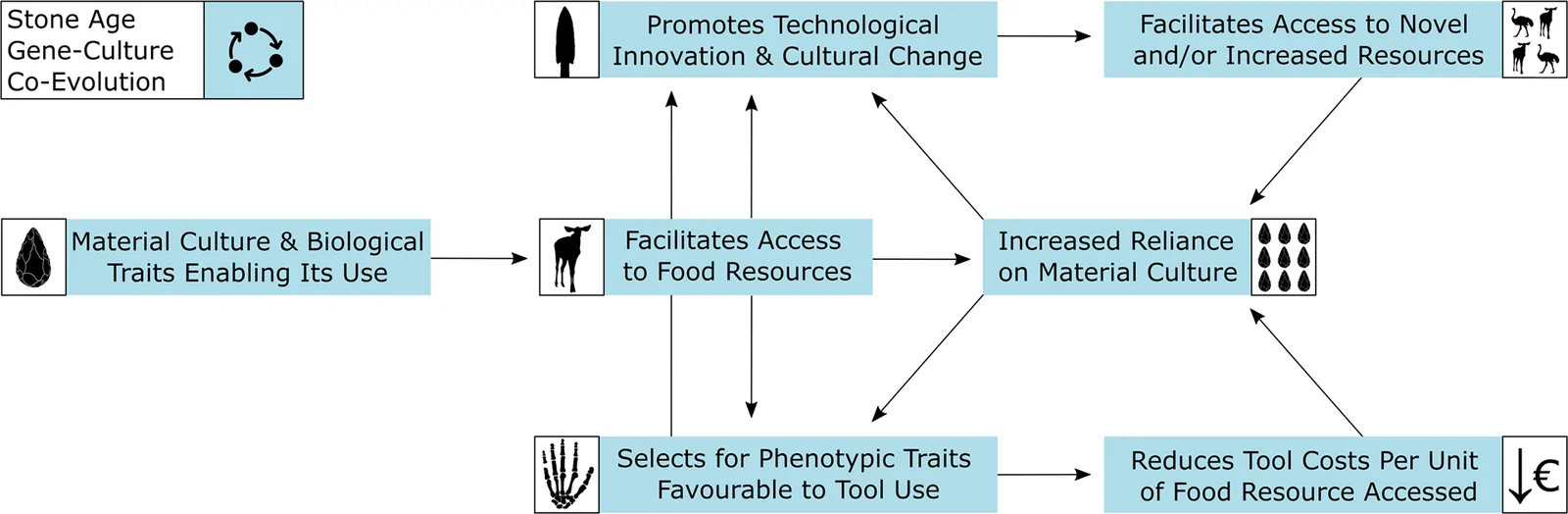
Generalised gene-culture co-evolutionary loop derived from Stone Age material culture applied to processing and/or acquiring food resources.

The persistence of lactase production beyond infancy is the best-known human GCC example, with the trait evolving alongside the spread of pastoralism in multiple independent populations [10]. Another well-known example is positive selection on the haemoglobin S (*HbS*) ‘sickle-cell’ allele (which provides protection against malaria) in Kwa-speaking West African agriculturalists, owing to crop-linked deforestation increasing mosquito prevalence [4]. These and other examples are, however, evidence of GCC subsequent to the advent of sedentary habitation and the *H. sapiens* agricultural niche [12,14].

The role of GCC in deep prehistory—the Stone Age of Africa and the Palaeolithic of Eurasia and the Americas—is more unclear [5,8,10,15], having been made obscure through populations being deeply removed from present-day societies [16], diverse taphonomic/search biases and the poor preservation of biomolecular evidence. Yet, we know a surprising amount about the material culture that is available, hominin fossil evidence is increasing exponentially (specimens and understanding), limited biomolecular evidence does exist and the use of data from extant referents is commonplace. By combining these lines of evidence, archaeologists and palaeoanthropologists have produced an at times detailed picture of GCC in the hominin lineage.

Here, current understanding of GCC in the Early and Middle Stone Age (ESA and MSA, respectively) is reviewed, and new hypotheses are outlined, to support a renewed research agenda focused on GCC's early—pre-agricultural—impact. By necessity, material culture is the focus here; principally stone tools are discussed, but when possible, organic technologies and other cultural traits are mentioned. Similarly, it focuses on the African fossil and archaeological record, given the Eurasian record is unique relative to Africa and *H. sapiens* originated in the latter (supplementary material, figures S1 and S2). Further, by the time *H. erectus s.l.* dispersed into Eurasia *ca* 1.8 Ma, many co-evolutionary relationships had likely been present for an extended period.

## 2. Plio-Pleistocene Stone Age culture

### (a) The Stone Age archaeological record

Artefactually evidenced hominin material culture—the archaeological record—is first observed *ca* 3.30 Ma at the stone tool site of Lomekwi 3 (Kenya) [17]. Lomekwi 3′s artefacts were likely used to process/acquire food resources given the intentional production of large (>10 cm) sharp-edged flakes suitable for cutting and percussive tools feasibly used for food extraction [17,18]. In turn, the ESA is inextricably linked to fitness-related behaviours from its outset [19]. This relationship continues throughout the Stone Age, including the two well-defined technological periods of the ESA: the Oldowan and Acheulean.

Oldowan archaeological sites, characterized by free-hand knapping of flakes from cores [20], are observed from *ca* 2.90 Ma at Nyayanga, Kenya [21]. Subsequently, three Ethiopian Oldowan sites are known from 2.75–2.57 Ma [22,23], and sites become more frequently observed through the next million years across Africa. Site frequencies through time follow a Weibull distribution, suggesting Oldowan tools became an increasingly practised behavioural trait [23,24]. The 2.44 Ma site of Ain Boucherit (Algeria) expands the Oldowan's spatial range to North Africa, while its presence at Kilombe's (Kenya) high-altitude 1.78 Ma caldera site attests to its use within diverse ecologies [25]. Lokalalei 2C (Kenya) evidences highly capable, controlled Oldowan flaking from at least 2.27 Ma [26]. Southern African Oldowan artefacts are first observed from 2.22 Ma [23]. West African sites remain largely undocumented, although they likely exist. Throughout the period, cut-marked faunal bones attest to flakes being used as butchery tools, but they likely also processed plant materials [18,20].

The African Acheulean is defined through the production of bifacially flaked, elongated core tools (hand-axes and cleavers) and the systematic production of large flakes (>10 cm) from cores [27]. Bifacial core flaking to create an acute edge suitable for cutting is first observed *ca* 1.75 Ma, with artefacts from Kenya, Ethiopia and Tanzania dating to 1.76–1.68 Ma [28]. Statistical estimates currently place the Acheulean's origin at 1.82 Ma [28]. As the Acheulean proceeds, new cultural traits emerge and accumulate [1], notably including the systematic manufacture of bone tools by 1.5 Ma [29], potential fire use at 1.5–1.0 Ma (but certainly by 780 ka [30]), barbed bone points by 800 ka [31], and wooden structures, digging sticks and spears by at least 400 ka [32] (supplementary material, figure S1). Throughout, faunal bones attest to increasing early-access exploitation of medium-to-large species [19,33]. An overall increased reliance on animal foods is contested [34]. Plant foods likely continued to be routinely exploited [18].

From *ca* 500 ka, systematic blade and point production may be present in southern Africa, but it is not widely observed until the MSA [35]. The production of hand-axe and cleaver flake blanks through Levallois-like (prepared core) methods is evidenced by 400 ka, but sequentially standardized/planned large flake manufacturing is present in Victoria West cores from *ca* 900 ka [36]. Levallois flaking can be observed from 320–280 ka in South Africa and Kenya [28]. The MSA broadly emerges from this point onwards, marking the onset of a technologically and culturally complex, and at times regionally dependent, archaeological record [35]. Southern Africa's Still Bay bifacial point industry (77–70 ka) [35] is a prime example.

Archaeological sites do, however, very rarely capture the true temporal and spatial range of past cultural phenomena. Sparse ESA-to-MSA records are particularly prone to such inaccuracies [28]. Stone tools were, therefore, almost certainly manufactured prior to Lomekwi 3.

### (b) Miocene and Pliocene hominin material culture

Parsimony suggests the earliest hominins were capable of relatively complex *Pan*-level tool-use and cultural behaviours (supplementary material, figure S1) [37–41]. Including diverse food-acquisition (i.e. fitness) related tool-use behaviours [39]. Some of these behaviours could have influenced, or been influenced by, hominin biological evolution from the late Miocene. Using a complete record of Oldowan sites [23], optimal linear estimation (OLE) modelling (following [42]) places the technology's emergence between 3 147 690 and 3 435 008 years ago (3.15–3.44 Ma). Dates that could place Lomekwi 3 within the Oldowan's temporal range. Irrespective, if Lomekwi 3 is combined with known Oldowan occurrences, the origin of intentionally manufactured flaked stone tools is estimated (via OLE) to occur in the mid-Pliocene, 3 887 525 years ago (3.89 Ma). These estimates are over-precise relative to ESA date error ranges, but they enable us to go beyond the known archaeological record in the pursuit of understanding Stone Age GCC.

Intentionally flaked stone artefacts do not, however, necessarily represent the start of hominin tool-related dependence. Gürbüz & Lycett [43] proposed that bone flakes could have served as a technological precursor, given their ability to provide sharp edges (solution) within meat-processing scenarios (fitness-related problem). Eren *et al.* [44] argue that ‘naturaliths’, defined as naturally produced sharp stone fragments, may also have formed part of the hominin toolkit prior to the advent of intentional stone flaking. Although difficult to identify in the archaeological record, if naturaliths and/or bone flakes contributed to hominin fitness they could feasibly have impacted hominin GCC trajectories from a very early point.

## 3. Which hominin species made which Stone Age artefacts?

Given the likelihood of flaked stone tools existing prior to 3.3 Ma, and following a speciose approach, *Australopithecus anamensis*, *Au. bahrelghazali*, early-to-mid *Au. afarensis* and *Au. africanus*, *Au. deyiremeda* and even *Ardipithecus ramidus* have potential to display evidence of GCC relationships with intentionally flaked stone tool technologies (supplementary material, figure S1) [45]. Similarly, from the earliest points in the hominin lineage, *Sahelanthropus tchadensis*, *Ar. kadabba* and *Orrorin tugenensis* could potentially have displayed GCC relationships with naturaliths, bone flakes or *Pan*-level modified and unmodified tool types.

Lomekwi 3 is commonly associated with *Kenyanthropus platyops* and late *Au. afarensis* [17], with the former more often assigned owing to their co-occurrence at West Turkana, Kenya. *Australopithecus afarensis* is, however, known to the north and south of Lomekwi, incorporating the site within the species's range [46,47]. *Paranthropus aethiopicus* is also known from the same geological context as the Lomekwi 3 tools (the Lomekwi Member) [48].

Up to 15 hominin taxa temporally overlap with the Oldowan [45]. In East Africa, the early-to-mid Oldowan is strongly associated with early *Homo* (*H. habilis* and *H. rudolfensis*), followed by late *Au. afarensis* (when using modelled first appearance dates [FADs]) and potentially *P. aethiopicus*. *Paranthropus* dentition is directly associated with the 2.9 Ma Nyayanga Oldowan site [21], reinforcing the genus' role. Similar contextual associations exist with early *Homo* and *P. boisei* at Olduvai Gorge, Tanzania [49]. In southern Africa, Oldowan technologies are most strongly associated with Early *Homo*, *Au. africanus* and *P. robustus*.

No artefacts have been found at South Africa's Malapa *Au. sediba* site, but the species likely made stone tools [50] and occurs at the Oldowan-Acheulean transition. Similarly, late *P. boisei*, *P. robustus*, *H. habilis* and *H. rudolfensis* all overlap with the early Acheulean but none are currently strongly associated with biface or large flake technologies. *Homo erectus* and *H. ergaster* (hereafter *H. erectus*) have for a long time [27] been associated with the Acheulean's origination and are near universally considered its sole manufacturer until later Middle Pleistocene (MP) *Homo* phenotypes emerge. From 900 ka, fossils assigned to *H. erectus* disappear and more derived, *H. sapiens*-like, attributes emerge under a range of species' names [51]. These MP populations produced later Acheulean occurrences until the emergence of *H. sapiens* and the MSA technologies our species is associated with. Species and technological transitions were, however, gradual and overlapping [28], and some basal *H. sapiens* could have produced Acheulean technologies. *Homo naledi* currently has no material culture associated with it. *Homo sapiens* is the only species associated with the Later Stone Age (LSA).

## 4. Hominin musculoskeletal anatomy

### (a) Hand

A fundamental tenet of GCC is that genetic variation can significantly impact a cultural trait's expression [4,12]. Within a Stone Age context, individuals with phenotypic traits favourable to stone tool use had the potential to gain tangible fitness-related benefits. It has repeatedly been demonstrated that present-day *H. sapiens* with high precision-grip force potential and longer digits use flake tools significantly more efficiently, and are exposed to lower joint contact stresses [52,53]. Moreover, individuals with larger hands and stronger precision grips use hand-axes more efficiently [53], demonstrating that population-level variation in manual traits relevant to stone tool use could have impacted hominin fitness.

The ability to forcefully perform precision grips has long been linked to ESA material culture [54]. Principally, our capacity to oppose the distal pollical phalanx (DPP) against the radial/palmar surfaces of the second distal and middle phalanges when securing flakes [54]. This requires a relatively high thumb-to-finger-length ratio, which is lacking in other extant great apes and *Ar. ramidus*, but likely present in the *Pan-Homo* last common ancestor (LCA), along with *Au. afarensis, P. boisei* and *Au. sediba* [50,55,56]. Combined with 1D : 2D ratio variation not significantly impacting *H. sapiens* stone tool-use [53], this trait potentially experienced no culturally linked selective pressures in early humans. Yet, a relatively long thumb is advantageous to precision gripping and the lack of this trait in *Pan* [55] helps explain their inefficient (not ineffective) precision grip [50,57], and potentially their, and *Ar. ramidus'*, non-adoption of flaked stone tool culture [40]. Conversely, high 1D : 2D ratios through most of the hominin lineage help explain how the earliest flake tool-use events (intentionally modified or naturaliths) provided benefits enough to make their social transmission worthwhile [23,24,58].

*Homo sapiens* also display a well-developed flexor pollicis longus (FPL). The muscle attaches to the DPP and facilitates its forceful flexion, which is essential when securing hammerstones, flake tools and stone cores [54,59]. A fully formed FPL has been suggested for Neanderthals (*H. neanderthalensis*) [50], and following the principle of parsimony African MP populations probably displayed a similar state. The FPL is absent in *Pan* [60], but probably present in *O. tugenensis* [55], and maybe present in *Ar. ramidus* [50]. When combined with a high thumb-to-finger ratio in the *Pan–Homo* LCA [55], the FPL was feasibly present in the very earliest hominins [60]. An independent FPL in early hominins could feasibly be linked to naturalith or *Pan*-level tool-use behaviours [50,54]. *Australopithecus afarensis*, *P. boisei*, *H. naledi* and other diminutive stone tool-linked hominins display a well-formed DPP FPL attachment site [50,56]. We have few manual fossils for MP hominins and *H. erectus* [50], so it is difficult to infer how the FPL evolved in response to Acheulean technologies. Given the high loading demands placed on the DPP during stone tool manufacture/use [59], it appears likely that even if an independent FPL was present in the *Pan-Homo* LCA, it could have experienced additional selective pressures during the ESA. Feasibly, hammerstone use—and therefore Oldowan flaking—may not have been possible (cf. [59]) until prior technologies selected for the ability to exert high DPP forces; additional research is required to support this hypothesis. A larger FPL would, in turn, have facilitated more efficient stone tool behaviours, further increasing hominin dependence and the strength of selective pressures acting on the muscle [53,59].

One of the few known *H. erectus* manual fossils is a 1.42 Ma third metacarpal [61]. It is significant for its large size [61]—suggesting an ability to effectively use large Acheulean tools [53]—but also for the presence of a styloid process, a trait previously only observed in *H. sapiens* and Neanderthals [50,54]. The styloid process stabilizes the third carpometacarpal joint against hyperextension and partial dislocation, suggesting its evolution in response to tool-derived loading demands [61]. Indeed, the percussive use of hammer-stones and the translating of stone cores heavily recruits distal and proximal portions of the third digit, respectively [59]. Once *Homo* was able to exert high loads against digits two-to-five, hominins were more effectively able to forcefully perform cradle grips, which are essential to hand-axe/cleaver use [54]. The Acheulean's emergence *ca* 1.8 Ma may, then, depend partially on the presence of a third metacarpal styloid process. Alternatively, the habitual use of large Acheulean tools may have exerted selective pressure such that the styloid process evolved in *H. erectus* by 1.42 Ma. This ‘chicken and egg’ (circular causality) scenario, which is common to GCC mechanisms, will be difficult to resolve without more detailed evidential frameworks. The cradle grip is also essential for platform preparation, a *ca* 500 ka Acheulean technological innovation that requires greater manual forces relative to any prior core handling activity [62]. This technological attribute may, therefore, have only originated after relatively *H. sapiens*-like manual anatomy evolved [62].

While manual fossils are infrequently discovered, they display numerous traits that inform on past interactions with material culture [50]. When combined with the uniqueness of our manual anatomy [54,55,60], it becomes clear that some of the best available evidence for early hominin GCC concerns the hand. No single trait, however, facilitated ESA tool use, and relevant features evolved in a mosaic [50] fashion alongside a similarly mosaic-like cultural trajectory that varied between species. For example, *P. boisei* displays an exceptionally broad apical tuft and well-developed thenar musculature [56], potentially revealing a greater reliance on precision-held flake tools relative to co-occurring species, including *H. erectus*. Combined with *H. erectus*’s Acheulean association, it supports the presence of distinct technical niches and highlights the need for species-specific GCC hypotheses. Figure S3 in the supplementary material outlines a possible scenario for ESA hand-tool GCC.

### (b) Cranium, mandible and dentition

Few cranial, mandibular or dental traits—beyond those linked to encephalization and language—are explicitly linked to hominin GCC mechanisms. Zink & Lieberman's [63] demonstration that processing meat and plant foods with stone tools prior to chewing significantly reduces masticatory demands is the only prominent example. With tooth size and enamel thickness broadly reducing through time in the *Australopithecus*-to-*Homo* lineage [63,64], the ability of lithic technologies to ‘free’ hominin dentition from excessive chewing cycles and forces arguably made this possible. Indeed, selection for smaller masticatory features could only have occurred if there were no strong dietary demands to retain large teeth and chewing muscles [63]. *Paranthropus* retained such demands [64] but seemingly simultaneously manufactured stone tools [45], suggesting a greater dietary reliance on their teeth, with stone tool use *potentially* being ancillary. Conversely, and following Zink & Lieberman [63], the smaller dentition observed in early-*Homo*-to-*H. erectus* suggests a comparatively greater tool reliance. Once smaller masticatory features were present, hominins could have become locked into pre-processing foods, further ingraining stone tool material culture in hominin populations [65].

With temporalis muscles reducing in size owing to reduced masticatory demands, hominin zygomatic arches and maxillary features become less robust, contributing to *Homo*’s comparatively small face. Speculatively, the lack of a sagittal crest in early *Homo* (although see KNM ER 1805)—the temporalis attachment point in *Paranthropus* and some *Australopithecus*—further reinforces the potential impact of a stone-tool-assisted diet on cranial anatomy. The cultural practice of controlled fire use may have also played a role in changing masticatory anatomy. Food toughness and fragment size can be reduced through exposure to high temperatures, and there is convincing evidence of some *Homo* populations cooking by *ca* 780 ka, but the consumption of thermally altered foods may have occurred much earlier [30]. As with stone-tool processing, and potentially acting alongside it, a co-evolutionary loop had potential to develop where cooking altered selective pressures allowing for more gracile masticatory anatomy, which led to a greater cooking reliance, which further altered pressures acting on masticatory anatomy (figure 1) [66].

### (c) Torso

#### (i) Intestinal tract

Coinciding with *H. erectus*’s larger brain and smaller dentition, we also observe a reduced intestinal tract size in hominins from *ca* 2.0 –1.8 Ma, albeit not to the *H. sapiens* condition [66]. Aiello & Wheeler [67] famously linked this smaller intestinal size to a higher quality diet, with both working to create or free up energy to fuel a larger, more costly brain (figure 2a). Central to their ‘expensive tissue hypothesis’ was the ability of *H. erectus* to increase its dietary quality by moving away from fibrous plants and towards nutrient- and energy-rich foods. To do this, it was necessary for *H. erectus* to perform ‘more complex foraging behaviour’ [67], which was fleetingly referred to in the original publication but is central to the hypothesis's position as an important Stone Age GCC example [13,15].

**Figure 2.**
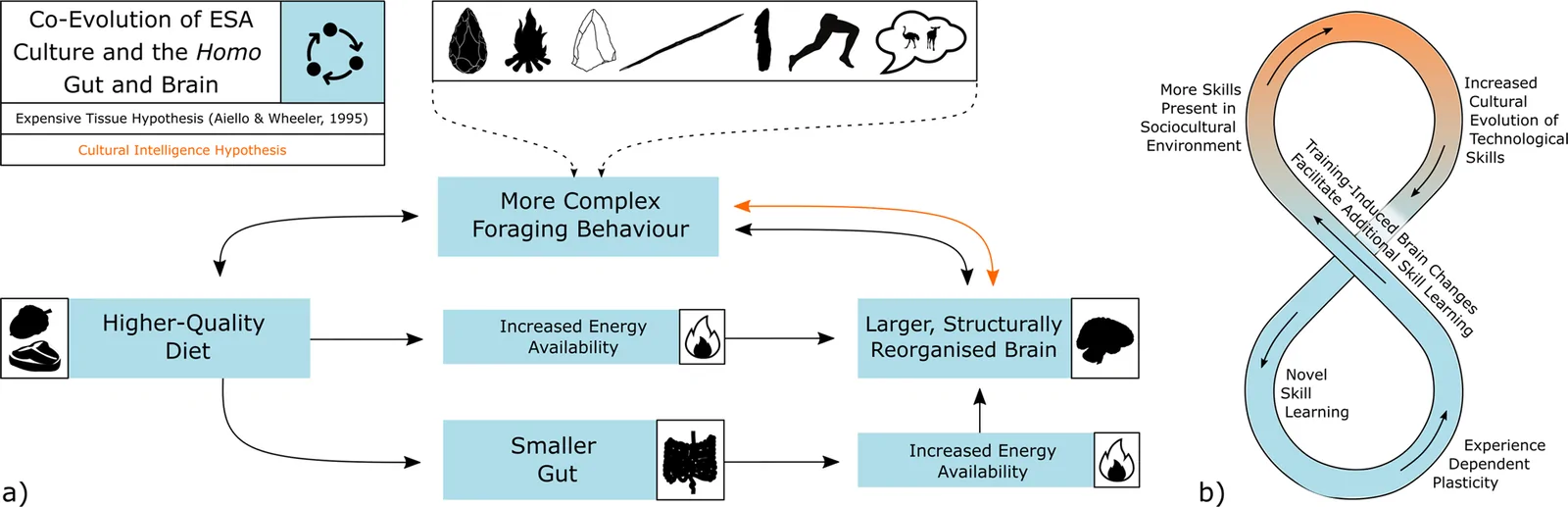
(a) The ‘expensive tissue hypothesis’ revised to emphasize the role of Stone Age culture. The bi-directional influence of ‘more complex foraging behaviour’ was not highlighted by Aiello & Wheeler [67]. (b) Hecht *et al.*’s [68] bio-cultural feedback loop between culture and brain structure.

As figures S1 and S2 in the supplementary material highlight, a series of important dietary-linked cultural innovations coincide with, or appear shortly after, *H. erectus*’s appearance. Their presence could have enabled *H. erectus* to shift its diet towards animal food resources or high quality plant foods. In turn, a co-evolutionary loop feasibly developed, whereby early technological innovations facilitated an initial move towards a higher-quality diet, which increased the energy available for a larger brain, which in turn facilitated the adoption of additional/more complex foraging behaviours (including technologies) that could further increase dietary quality (figure 2a). Behavioural elements not linked to material culture also probably contributed to this dietary shift, but are underinvestigated. Many of these, such as persistence and complex ambush hunting, could have been socially learnt [69,70].

#### (ii) Thoracic cavity

If cardiovascularly intense fitness-related behaviours can be linked to socially learnt information, such as during coordinated persistence hunting, swimming/aquatic food collection or long-distance raw material transportation [69,71,72], GCC relationships could feasibly have existed with hominin respiratory functional capacity. Indeed, culturally mediated cardiovascularly intense behaviours could have created selective pressures favouring more effective respiratory cycles and gas exchange. Individuals displaying favourable respiratory capacities would, in turn, have more efficiently performed these behaviours (gained a fitness advantage), increasingly practised/shared relevant information and prompted novel cultural traits to emerge; behavioural dependence and selective pressure strength could subsequently have increased (note that data supporting this feedback loop is required). In *H. erectus*, their relatively wide, short and deep rib cage, which was derived relative to earlier species [73], supports these pressures having already impacted the hominin phenotype. Hints of *Homo*-like upper thoracic expansion have, however, also been observed in *Au. afarensis* [74], but not *Au. sediba* [75]. With *H. sapiens* displaying a highly derived, efficient capacity for intense cardiovascular behaviours [73], cardiovascular GCC arguments could be maintained into the MSA.

#### (iii) Shoulder

Non-human great apes routinely throw objects, including during potential cultural traditions, but usually do so underarm, over short distances, and with poor aim [39]. By contrast, *H. sapiens* throw accurately, forcefully and overarm during multiple fitness-related and culturally mediated activities (e.g. hunting, defence, display and play). Underpinning the ease with which we accurately accelerate projectiles is our derived shoulder anatomy [76]. Pelvic, vertebral, humeral, carpal and costal anatomy all contribute [77,78], but our shoulder absorbs energy created through the thorax and redirects it down the arm, ultimately allowing object acceleration to peak at the point of manual release [77,78]. Pliocene hominins and Pleistocene *Australopithecus* and *Paranthropus* display relatively primitive shoulder anatomy, suggesting the retention of arboreal functionality [79,80]. Not until *H. erectus* is there major structural reorganization in the hominin shoulder, with clavicle/scapular orientation and form approaching or falling within *H. sapiens* ranges [76]. Suggesting if GCC underpins later *Homo* shoulder anatomy, it is most likely linked to early-to-mid-Pleistocene culture.

Foundation for a throwing-derived GCC mechanism may lie in early hominin defensive behaviours. Building on a *Pan* baseline [39], if unmodified projectiles (e.g. sticks and stones) could protect carcasses from scavengers [77], defend against competing hominin groups and predators or aid reproductive success (female selection *or* male–male competition), those with anatomy facilitating more forceful or precise aims could have had an advantage. Species that did not experience strong arboreal locomotory pressures would have experienced more rapid evolutionary change [79,80]. More speculatively, once *defensively* routine, and potentially with some derived anatomy, novel *offensive* throwing behaviours could have emerged, become socially learnt and adaptively important and increased the strength of evolutionary pressures acting on shoulder anatomy. For example, throwing objects to split large ungulates from their offspring could have significantly aided early hunting. More effective throwing could also have facilitated riskier versions of earlier behaviours: actively stealing carcasses from other scavengers/predators, for example, or more regularly encroaching into the territories of other hominin groups.

A reliance on, and the spread of, such activities alongside new anatomical capabilities could in turn have driven cultural evolution and technological innovation. The effective throwing of non-modified sticks, for example, could have borne more complex, task-specific modifications and design intent, including those relating to flight aerodynamics. Ultimately, the efficacy of wooden-projectile use would increase, potentially leading to greater task success, increased reliance on material culture, and even stronger selective pressures (again, these mechanisms are currently based on logical conjecture). Modified wooden spears are observed in the archaeological record from *ca* 400 ka, but based on this GCC argument and *H. erectus* anatomy [77], it would be reasonable to infer their Early Pleistocene origin.

### (d) Lower limb

The evolution of obligate bipedalism in humans has rarely been described through GCC mechanisms. Principally this is because we see clear bipedal adaptations in *Au. afarensis, Paranthropus* and *Au. anamensis* [81,82], suggested mosaic bipedal features in *S. tchadensis*, *Ar. ramidus* and *O. tugenensis* [83–85], and bipedal hominin footprints 3.66 Ma at Laetoli, Tanzania [86], and all occur before the 2.6 Ma threshold that, prior to 2015, marked the earliest flaked stone tools [22]. Partially for this reason, bipedalism is principally hypothesized to have evolved in response to arboreal orthograde clambering behaviours and the occupation of increasingly fragmented forest-to-woodland habitats [82,87–89]. *Au. sediba*, *Au. africanus* and early *Homo* display some arboreal locomotory structures alongside derived features linked to obligate bipedalism [81,90], but by the arrival of the Acheulean *ca* 1.8 Ma, *H. erectus* appears to have *H. sapiens*-like locomotory anatomy [66].

If hominin culture and lower limb anatomy influenced each other in a co-evolutionary relationship (figure 3), relevant mechanisms could, then, have had a Miocene-to-Pliocene origin, *or*, enacted pressure during the Pliocene-to-Early Pleistocene after hominins evolved bipedal efficacy in response to other processes. A Miocene-to-Early Pliocene origin could mean naturaliths had an impact. It is difficult to envisage their transportation during quadrupedal locomotion, be it arboreally or terrestrially, particularly over long distances. Non-optimal manual postures would have been required, and external forces would have resulted in lacerations. Hence, the bonobo (*Pan paniscus*) Kanzi transported individual stone flakes in his mouth (e.g. [91]). Thus, if naturaliths were adaptively important, those able to more efficiently bipedally transport them would have had an advantage. Subsequent generations could have exhibited derived anatomy and displayed a greater reliance on these (and subsequent) tool types. If bone flakes were a precursor to flaked lithics, the above hypothesis can be repeated. Both theoretical scenarios help to explain why we see long-distance material transportation at very early lithic archaeological sites [71].

**Figure 3.**
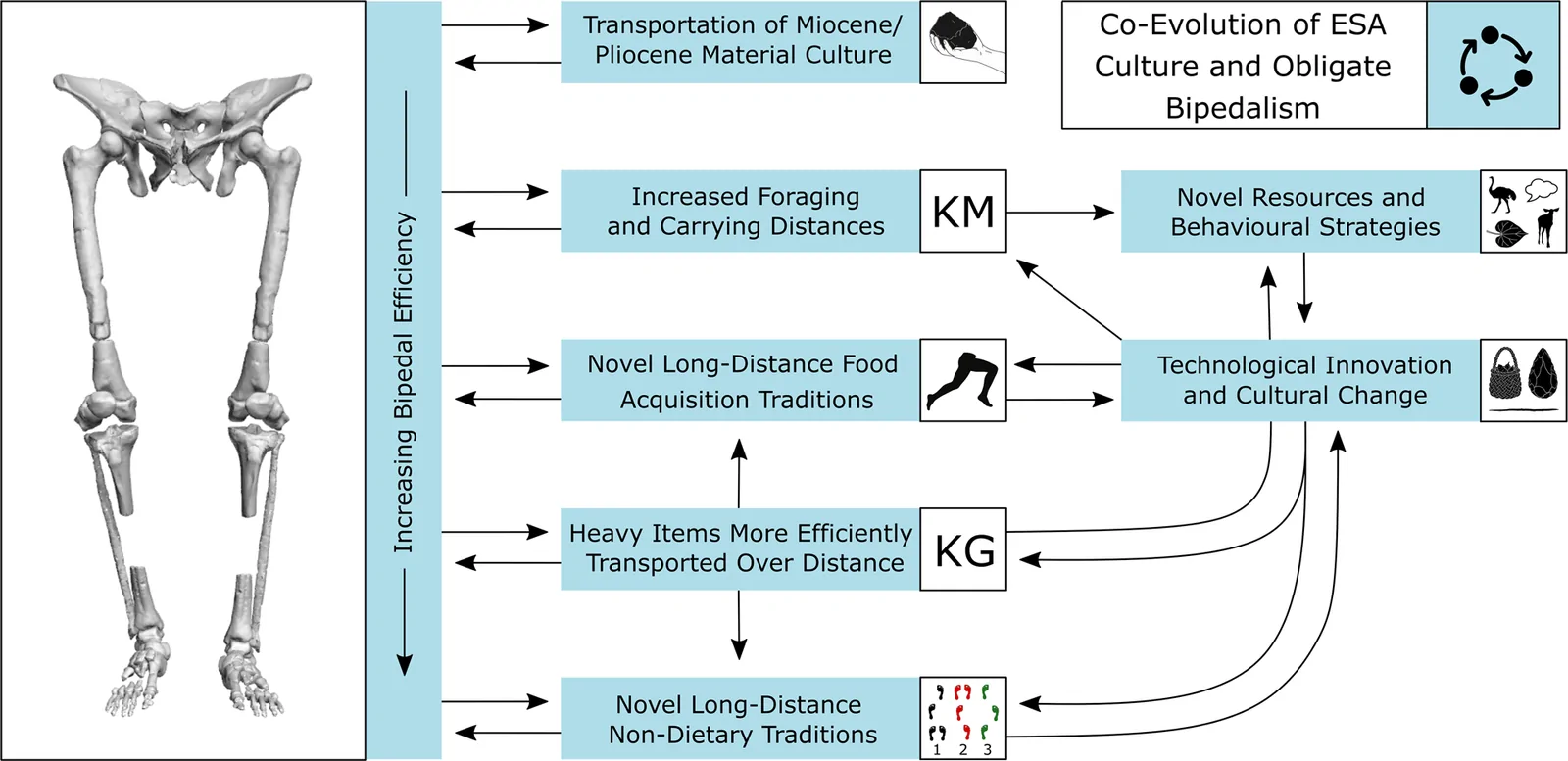
Theoretical scenario for the co-evolution of Stone Age culture and anatomical traits facilitating bipedalism, based on logical conjecture.

Carrying objects other than cutting tools may also have played a role [87,91]. Comparative observations with *Pan* [92,93] and modern humans [94] demonstrate that carrying underpins numerous fitness-linked behaviours. Many of these behaviours were feasibly practised by Miocene-to-Pleistocene hominins within a *Pan*-like adaptive grade. Although substantive wooden artefacts are not observed until the MP [32], parsimony suggests large portions of plant material could have been transported as part of early socially learnt behaviours [39]. The transportation of food resources is another commonly referenced pressure [88,92]. There are diverse scenarios where this could have occurred culturally (e.g. nut cracking, coordinated hunting and cooperative honey collection), with subsequent generations feasibly practising cultural variants linked to their derived anatomy. Even traditions pertaining to infant carrying or non-dietary social provisioning could have had a GCC role [88,93].

Bipedalism-linked GCC mechanisms also possibly extended beyond carrying. Sharp flake tools are routinely linked to processing terrestrially sourced animal foods, and both *H. sapiens* and chimpanzee populations display differences in socially learnt foraging (including hunting) behaviours [41,95]. Individuals whose anatomy facilitated more efficient sourcing of these unpredictable resources would have had an advantage; again, potentially contributing to a feedback loop where increased cultural reliance leads to cultural change, heightened resource dependence and subsequent intensification of biological/cultural selective pressures (figure 1). In fragmented, woodland-like habitats, the adaptive value of efficient bipedal locomotion is well established [88]. In forests, efficient bipedal locomotion could have been important within a *Pan*-level foraging grade [89], but especially if there was a need to follow animal tracks, a skill which is itself socially learnt in *H. sapiens* [95]. Potentially, scavenging and some types of forest foraging *could* have been less efficient from elevated, arboreal positions.

With the Acheulean defined by large cutting tools, the technology feasibly did not emerge until hominin bipedal locomotion was efficient enough to transport heavy objects over long distances at low cost (figure 3). The co-emergence of Acheulean tools and *H. erectus* lower limb anatomy lends support to this hypothesis. Their co-dependence may even explain why the less bipedally efficient *P. boisei* potentially only manufactured Oldowan tools [45]. Similarly, Oldowan and Lomekwi-3 style tools may not have emerged, or remained an occasional energetically expensive behaviour, until cores could be efficiently bipedally transported. Again, the fossil record broadly aligns with this scenario as obligate, but not highly efficient, bipedalism is observed in early *Homo*, *Paranthropus* and *Au. afarensis*. Yet, if tool transportation was itself a selective pressure, this raises another circular causality scenario in need of evidence-led appraisal.

None of the above scenarios is necessarily more likely or influential than another. They may even have interacted and/or led to one another, or not existed at all (figure 3). What is clear is that bipedalism could have been selected for through multiple fitness-related traditions and acted to create—and indeed acted on—cultural variation, at very early points in the hominin lineage. These relationships would not necessarily have left an archaeological signature.

## 5. Hominin cognition, learning and language

Hominin cognitive and language capabilities appear to have evolved in unison and represent the most heavily investigated aspect of Stone Age GCC. To the extent that a comprehensive review is not possible here. Stout [24,96], Sterelny [97], Ekström *et al.* [65] and Barham & Everett [98], among others, discuss how Stone Age culture impacted, and depended on, hominin brain and vocal tract evolution. Key relationships are outlined here.

Diet and ecology are key drivers of primate brain size [99,100]. Yet, *H. sapiens* and other MP hominin cranial capacities are extreme outliers among apes [101], and while dietary and ecological factors are important (e.g. [67,102]), they cannot alone explain hominin cognitive trends. Hominin cranial capacities increase through time, as a broad across-genus trend (with outliers [103,104]), but also within species [105]. The latter suggests encephalization was driven by multiple pressures acting independently on individual species. Moreover, different species display different temporal trends, attesting to varying strengths of selective pressure [105]. We also see shifts in brain shape and cortical organization, with derived frontal lobe features not emerging until after 1.7 Ma with *H. erectus* [106] and *H. sapiens*-like features appearing in the MP [107]. This is important, as the Broca's area, which is strongly associated with speech production and language comprehension, is located in the inferior frontal gyrus, supporting expansion of hominin language capabilities in *H. erectus,* and again in *H. heidelbergensis* [106,107].

Complex culture is thought to have contributed to non-human great ape cranial capacity size via the partially GCC-dependent ‘cultural intelligence hypothesis’ [11]. Similar mechanisms may underpin hominin encephalization. Will *et al.* [102] found MP *Homo* in areas of low net primary productivity (steppe and grasslands) to display larger brains, which they ascribe to more frequent and complex hunting of large bovids. This suggests larger brains facilitated more effective and complex hunting strategies, which in turn supported larger brains, as partially outlined by Aiello & Wheeler [67] (figure 2a). The same hypothesis can be applied to early Pleistocene hominins, including via complex scavenging behaviours, albeit with reduced carnivory rates [34], and in turn, selective pressure strength, helping to explain shallow encephalization slopes in *H. erectus* and *Au. afrensis* [105]. Language-specific components of this hypothesis also exist. A sliding scale between hunting complexity and the presence of language can be inferred; the more complex, sequential and cooperative the strategy, the more likely its success depended on complex language, with a feedback loop between the two supporting their respective evolutionary trajectories (figure 2a) [108]. A mechanism potentially built on earlier requirements to flexibly process structured, sequential information [109]. Related arguments exist where substantial hunting yields are linked to large, complex social structures [33], from which similar feedback loops can be inferred. Plio-Pleistocene hominins may even have become pre-adapted for speech through the dietary GCC selection of gracile facial anatomy [65].

Plio-Pleistocene hominin culture, however, extends beyond hunting. The inception and sustained practice of knapping likely instigated learning costs and, subsequently, pressures favouring its effective social transmission [110]. From a *Pan*-like baseline, more effective emulation, imitation and other non-verbal teaching (e.g. gestural) abilities could have come under pressure from an early point [24,37,110,111]. Morgan *et al.* [58] demonstrate stone tool-making skill transmission to be aided by active teaching (particularly language-based), suggesting stone tool-reliant populations experienced pressures favouring complex verbal communication. Subsequent studies also stress the importance of gesture [111]. In turn, more effective learning and complex verbal communication could have aided cultural dissemination, promoted innovation and reduced copying error rates [110]. Potentially, the cultural ‘stability’ observed in Oldowan and Acheulean stone-tool culture was underpinned by effective social transmission methods [98,110]. With Acheulean technologies being more complex than the preceding Oldowan [112] and requiring extended periods of learning [113], language may have been a prerequisite for the former to emerge [58]. This raises the possibility of a language-capable, Acheulean manufacturing *H. erectus* coinciding alongside a non-language-capable, Oldowan manufacturing *P. boisei*.

Evidence suggesting stone tool-language co-evolution is also found in paleoneurological research. Stout *et al.* [112] demonstrate the ‘action grammars’ of later Acheulean tool-making to be more complex than those required for Oldowan-level flaking. With language underpinned by grammatical sequences, and language complexity linked to sequence complexity, the Pleistocene co-evolution of both abilities appears feasible. Potentially, selective pressures acting on one reciprocally aided advances in the other [108]. Neuroimaging work supports this inference; the manufacture of Acheulean technologies activates regions of the brain strongly linked to language acquisition/capabilities and sensorimotor/visuomotor control [114,115]. The feasibility of natural selection acting on neurophenotypic variants associated with tool making was demonstrated by Hecht *et al.* [68], who revealed modern *H. sapiens* with increased white matter fractional anisotropy beneath the left inferior frontal gyrus to more capably manufacture Acheulean tools. Moreover, during stone tool-making training, structural changes in areas connecting inferior parietal and inferior frontal cortices were observed [68]. Meaning not only were stone tool-manufacturing derived selective mechanisms possible, but learning new Stone Age technologies could have ‘exerted plastic effects on brain anatomy that enhanced technological learning capacities and thus facilitated further cultural evolution’ [68], feeding into a biocultural feedback loop (figure 2b). Through the perceptual-motor hypothesis, *Pan*-level technological capabilities feasibly laid the foundations for language-based GCC loops to develop [96], supporting a deeply rooted origin for tool-cognitive-learning-language GCC in the hominin lineage [37].

Recurrent within language and cognition debates is the role of cumulative culture as a marker of advanced ability [65,97]. With Paige & Perreault [1] artefactually evidencing hominin dependence on cumulative culture by *ca* 600 ka, it attests to a later than anatomically expected (see above) origin of complex language in *Homo*. In turn, linking the innovation of later Acheulean technologies to complex language. This aligns with palaeoneurological data evidencing structural and encephalization shifts in *Homo* during the second half of the MP, with Bruner [107] proposing an extended cognition theory integrating ‘brain, body and technology’ as part of a single co-evolutionary unit. A concept that builds on prior human-technological system optimization theory [15] to explicitly include core cognitive functioning (e.g. motor control and working memory) and our excellent respiratory motor control, which, alongside our tongue and supralaryngeal vocal tract, underpins human speech [116]. Whether GCC contributed to the evolution, or maintenance, of relatively small brains in some hominins is unknown [117].

## 6. Discussion and conclusion

Coarse by necessity and limited to major anatomical (biological) elements, this review has nonetheless highlighted the breadth of routes through which GCC potentially impacted hominin Miocene-to-Pleistocene cultural and biological trajectories. Some research areas already have deeply embedded GCC concepts (e.g. cognition and tool size), but greater precision in our understanding is required. Other areas have substantial room for development, and this review highlights the need for more research. Some highlighted relationships may be further supported, others may be refuted with traits linked to alternative mechanisms, but without testable hypotheses and a research agenda directed towards the Stone Age (and earlier), the role of GCC in our evolutionary history will not be fully understood.

A lack of Stone Age ancient DNA means there is little to no insight into how culture interacted with genotypic variation of the type underpinning some modern *H. sapiens* GCC relationships [4,12]. Further, some types of culture cannot be discussed owing to a lack of evidence. For example, sex-biased, potentially culturally derived, interbreeding patterns are evidenced between Neanderthals and *H. sapiens* [118]. It is plausible that culturally linked interbreeding patterns impacted population trajectories in Africa, yet we have limited data to investigate this possibility. Other cultural traits that feasibly underpinned GCC mechanisms but could not be mentioned include: clothing (buffered against climatic extremes), culturally-mediated social displays (canine crown-size reductions), and frequent knapping injuries (impacting individual fitness). GCC likely had greater routes for influence as culture became more complex and diverse in some lineages, making its impact unevenly experienced between different species/populations.

Ultimately, many of these co-evolutionary trajectories contributed to *H. sapiens* and other MP hominin species constructing their own cultural niches [6,12,14]. This is not necessarily the case for all Stone Age populations, but by outlining how early GCC mechanisms led to some species' cultural dependence, it evidences a strength of impact across a breadth of bio-cultural traits not observed in post-agricultural GCC mechanisms. For example, humans evolved lactase persistence in response to GCC pressures, but populations were unlikely to have been entirely dependent on milk products for their survival. By contrast, the human hand—as one example—is arguably specialized for tool use, and our survival typically depends on manual technology-led food-procurement strategies. In sum, this review agrees with Laland [10], who suggested ‘the past 2 million years of human evolution may … have been dominated by gene-culture coevolution’, but with one caveat: 2 million years appears far too brief a timeframe.

## Supplementary Material



## Data Availability

Supplementary material is available online [119].
