# Supplementary material for: The co-evolution of hominins and Stone Age culture
Source: Proc Biol Sci. 2026 Oct 14;293(2080):20260772. doi: 10.1098/rspb.2026.0772 (PMC13678621; doi:10.1098/rspb.2026.0772)
Supplement: Supplementary file 1 [file rspb.2026.0772.zip › rspb.2026.0772/rspb20260772_si_001.docx]

**Supplementary Figures:** Proceedings of the Royal Society B

The Co-Evolution of Hominins and Stone Age Culture (doi:10.1098/rspb. 2026-0772)

**Supplementary Figure 1:** Hominin species’ temporal presence in Africa alongside important Early Stone Age (ESA) sites. Middle Stone Age (MSA) and Later Stone Age (LSA) cultural diversity is not well represented here.

**Supplementary Figure 2:** Hominin species’ temporal presence in Eurasia alongside important Lower, Middle and Upper Palaeolithic sites.

**Supplementary Figure 3:** A hypothetical scenario for the co-evolution of hominin manual anatomy and Early Stone Age culture based on current evidence. The complexity of the hand and cultural record makes for an extended, intricate GCC relationship that could only partially be detailed here.
